# Intestinal Homeostasis and Its Regulatory Role in Animal Growth and Development

**DOI:** 10.3390/vetsci13090965

**Published:** 2026-09-15

**Authors:** Penggang Liu, Shifeng Pan, Liangli Song

**Affiliations:** 1 College of Veterinary Medicine, Yangzhou University, Yangzhou 225009, China; 2 College of Animal Science & Technology, Ningxia University, Yinchuan 750021, China

The gastrointestinal tract is a highly dynamic biological interface, responsible not only for nutrient digestion and absorption but also for maintaining microbial, epithelial, immune, and metabolic homeostasis. Intestinal homeostasis depends on coordinated interactions among the gut microbiota, epithelial barrier, mucosal immune system, dietary components, and host metabolic signaling. Disruption of these interactions can impair nutrient utilization, compromise barrier integrity, promote inflammation, and ultimately affect animal growth, health, and productivity [1,2,3]. Accordingly, understanding the mechanisms that maintain intestinal homeostasis and identifying practical strategies to regulate these processes have become increasingly important in veterinary medicine and animal science.

This Special Issue, “Intestinal Homeostasis and Its Regulatory Role in Animal Growth and Development”, was established to bring together recent advances in this rapidly developing field. The contributions to this Special Issue address intestinal homeostasis from complementary perspectives, including microbiota modulation, probiotic interventions, nutritional regulation, intestinal barrier integrity, pathogen-associated dysbiosis, host–microbe interactions, inflammation, and systemic metabolic regulation. Collectively, these studies illustrate that intestinal homeostasis is not a single biological process, but rather an integrated network linking microbial ecology with host physiology and animal performance.

One major theme highlighted in this Special Issue is the potential of microbiota-targeted interventions to support intestinal health and animal performance. Essa et al. evaluated *Bacteroides fragilis* and *Enterococcus faecium* isolated from healthy sheep as probiotic candidates and demonstrated their ability to influence growth-related parameters, intestinal morphology, and gut microbial composition in mice. Wang et al. further characterized breed-associated differences in the ruminal microbiota and evaluated a Duolang sheep-derived *Ligilactobacillus salivarius* strain, highlighting the potential value of host-adapted microorganisms for modulating mucosal immunity and metabolic status in ruminants.

The effects of probiotic modulation may also extend beyond the gastrointestinal tract. Song et al. demonstrated that Lactobacillus and Bacillus supplementation improved laying performance in Zhedong White geese through distinct microbiota–metabolite–endocrine regulatory pathways. Duan et al. used integrated multi-omics to characterize gut microbiota–ovarian crosstalk in high- and low-laying Muscovy ducks, further linking microbial and metabolic variation with reproductive performance. These findings provide an important example of how changes in intestinal microbial communities and metabolic profiles may influence reproductive performance. In companion animals, Morua et al. investigated a combination of Aloe vera, Artemisia annua, and Curcuma longa in healthy dogs and observed changes in microbial taxa and predicted microbial functions associated with intestinal health. Together, these studies support the development of species-specific microbiota-oriented approaches while also emphasizing the need for further functional and long-term validation.

A second important theme concerns nutritional regulation and intestinal barrier integrity. Çayan and Coşkun evaluated dietary supplementation with sage (*Salvia officinalis* L.) leaf powder in broilers and reported improvements in growth performance and intestinal histomorphology, supporting the potential application of phytogenic feed additives as nutritional tools for promoting intestinal health. Tóth et al. reviewed β-casein polymorphism as a potential evolutionary trade-off and discussed the implications of A1 β-casein for gastrointestinal tolerance and agroecological resilience. Zhang et al. investigated N-acetylneuraminic acid in a piglet model and demonstrated that dietary supplementation altered intestinal microbial composition and microbial metabolic activity, including changes in formate and acetate production. These findings illustrate how specific nutritional components can reshape the intestinal ecosystem and potentially influence host physiology.

Beyond microbial composition, the physical integrity of the intestinal epithelium remains a central component of homeostasis. Zhang et al. reviewed the roles of tight junction proteins in weaned piglets, emphasizing their importance in regulating intestinal permeability, nutrient absorption, immune responses, and interactions between the host and intestinal microbiota. This work underscores the importance of integrating measurements of microbial composition with functional indicators of epithelial barrier integrity when evaluating intestinal health.

The contributions to this Special Issue also demonstrate how infectious challenges can disturb intestinal microbial and barrier homeostasis. Essa et al. showed that *Bacteroides thetaiotaomicron* BT6 alleviated the adverse effects of *Escherichia coli* O157:H7 challenge in mice, with improvements in microbial balance, intestinal barrier integrity, inflammatory responses, and oxidative status. This study illustrates the potential of targeted microbial interventions not only for maintaining intestinal health under normal conditions but also for restoring homeostasis following pathogen-associated disruption.

In neonatal dairy calves infected with *Cryptosporidium parvum*, Yachida et al. examined associations among milk management, intestinal microbial diversity, predicted microbial functions, and diarrheal status. Their findings highlight the complex interaction between early-life nutrition, enteric infection, and microbiome development. Similarly, Chen et al. demonstrated that *Histomonas meleagridis* infection substantially altered the cecal microbiota of chickens, reducing microbial diversity and shifting the relative abundance of beneficial and potentially pathogenic bacterial populations. Together, these studies show that intestinal microbial disturbances may represent both a consequence of infection and an important component of disease-associated physiological changes.

At the cellular and molecular levels, homeostatic mechanisms extend beyond microbial composition alone. Fu et al. reviewed the dual role of autophagy during viral infection, describing how this highly conserved cellular process can participate in antiviral defense while also being exploited by viruses to facilitate replication and immune evasion. Although autophagy operates across multiple tissues and cell types, its central role in cellular quality control, metabolism, and immune regulation provides a broader mechanistic framework for understanding host–microbe interactions.

Inflammation, oxidative stress, epithelial injury, and microbial dysbiosis are also tightly interconnected in intestinal disease. Jin et al. reviewed recent advances in natural products for colitis and summarized their potential effects on inflammatory signaling, oxidative stress, intestinal barrier integrity, immune regulation, gut microbiota, and programmed cell death. Importantly, this review also highlights key translational challenges, including insufficient mechanistic validation, variable bioavailability, limited pharmacokinetic information, and the need for standardized efficacy and safety assessment. These considerations are particularly relevant when translating promising experimental interventions into practical veterinary or nutritional applications.

An increasingly important concept emerging from intestinal research is that gut homeostasis should be considered within the broader context of inter-organ communication and systemic metabolism. Kuang et al. reviewed plant-derived exosome-like nanovesicles as emerging candidates for metabolic dysfunction-associated steatotic liver disease and discussed their potential roles in lipid metabolism, inflammatory regulation, oxidative stress, and gut–liver axis communication. Such approaches illustrate the growing interest in biologically derived nanoscale mediators as tools for modifying metabolic and intestinal–hepatic interactions.

At a broader level of organismal homeostasis, Tang et al. employed integrated metabolomic and transcriptomic analyses to characterize heat stress-induced changes in porcine granulosa cells. Their results revealed substantial metabolic reprogramming, particularly involving lipid metabolism and hormone-related pathways. This work complements the gut-centered studies in the Special Issue by illustrating how environmental stress can affect metabolic and reproductive homeostasis at distant tissue sites. Together with the microbiota–metabolite–endocrine relationships demonstrated in other contributions, these findings reinforce the importance of considering intestinal, metabolic, reproductive, and environmental factors as interconnected components of animal growth and development.

Taken together, the studies included in this Special Issue support a conceptual framework in which intestinal homeostasis emerges from interactions among microbial communities, epithelial barriers, immune responses, nutrition, metabolism, infectious agents, and environmental factors. Importantly, the consequences of intestinal regulation are not necessarily confined to the gastrointestinal tract. Through microbial metabolites, immune mediators, endocrine pathways, and inter-organ communication, changes originating within the intestinal environment may influence growth, reproduction, metabolic health, disease resistance, and overall animal productivity.

Several important directions deserve further attention. First, although microbiome profiling has provided extensive information on associations between microbial communities and host phenotypes, future studies should increasingly distinguish correlation from causation through microbial transplantation, defined microbial consortia, metabolite supplementation, receptor- or pathway-level validation, and functional rescue experiments. Second, inter-individual and interspecies variability remains a major challenge. Breed, age, diet, housing, environmental exposure, developmental stage, and baseline microbial composition can all influence the response to microbiota-targeted interventions. Standardized and longitudinal experimental designs will therefore be essential for identifying robust and reproducible biological relationships.

Third, the integration of metagenomics with metabolomics, transcriptomics, proteomics, and detailed host phenotyping will be important for moving beyond descriptive microbial profiling toward a mechanistic understanding of microbiota–host communication. Particular attention should be paid to microbial metabolites and host signaling pathways that directly connect intestinal changes with systemic physiological outcomes. Finally, promising probiotics, phytogenic compounds, natural products, bioactive metabolites, and other microbiota-targeted strategies require validation under practical production or clinical conditions, with a careful assessment of their efficacy, safety, dosage, duration, host specificity, and long-term stability.

In conclusion, this Special Issue highlights the growing importance of intestinal homeostasis as a central component of animal health, growth, and development. The collected contributions demonstrate that maintenance of a stable and functionally balanced intestinal environment requires coordinated regulation at microbial, epithelial, immune, nutritional, metabolic, and systemic levels. Continued investigation of these interconnected processes will support the development of more precise nutritional strategies, microbiota-based interventions, and disease-control approaches, ultimately contributing to improved animal health, welfare, production efficiency, and sustainable livestock and veterinary systems.

We would like to sincerely thank all authors who contributed their valuable work to this Special Issue and all reviewers for their time, expertise, and constructive comments. 

## References

[1] Bergstrom K.S.B., Xia L. (2013). Mucin-type O-glycans and their roles in intestinal homeostasis. Glycobiology.

[2] Zhang Z., Tanaka I., Pan Z., Ernst P.B., Kiyono H., Kurashima Y. (2022). Intestinal homeostasis and inflammation: Gut microbiota at the crossroads of pancreas–intestinal barrier axis. Eur. J. Immunol..

[3] Khasanah H., Kusbianto D.E., Purnamasari L., Dela Cruz J.F., Widianingrum D.C., Hwang S.G. (2024). Modulation of chicken gut microbiota for enhanced productivity and health: A review. Vet. World.

